# Age as a Determinant for Dissemination of Seasonal and Pandemic Influenza: An Open Cohort Study of Influenza Outbreaks in Östergötland County, Sweden

**DOI:** 10.1371/journal.pone.0031746

**Published:** 2012-02-23

**Authors:** Toomas Timpka, Olle Eriksson, Armin Spreco, Elin A. Gursky, Magnus Strömgren, Einar Holm, Joakim Ekberg, Örjan Dahlström, Lars Valter, Henrik Eriksson

**Affiliations:** 1 Department of Public Health, Östergötland County Council, Linköping, Sweden; 2 Department of Medical and Health Sciences, Linköpings Universitet, Linköping, Sweden; 3 Department of Computer and Information Science, Linköpings Universitet, Linköping, Sweden; 4 National Strategies Support Directorate, ANSER/Analytic Services Inc, Arlington, Virginia, United States of America; 5 Department of Social and Economic Geography, Umeå University, Umeå, Sweden; 6 Linnaeus Centre HEAD, Swedish Institute for Disability Research, Department of Behavioural Sciences, Linköping University, Linköping, Sweden; INSERM & Universite Pierre et Marie Curie, France

## Abstract

An understanding of the occurrence and comparative timing of influenza infections in different age groups is important for developing community response and disease control measures. This study uses data from a Scandinavian county (population 427.000) to investigate whether age was a determinant for being diagnosed with influenza 2005–2010 and to examine if age was associated with case timing during outbreaks. Aggregated demographic data were collected from Statistics Sweden, while influenza case data were collected from a county-wide electronic health record system. A logistic regression analysis was used to explore whether case risk was associated with age and outbreak. An analysis of variance was used to explore whether day for diagnosis was also associated to age and outbreak. The clinical case data were validated against case data from microbiological laboratories during one control year. The proportion of cases from the age groups 10–19 (p<0.001) and 20–29 years old (p<0.01) were found to be larger during the A pH1N1 outbreak in 2009 than during the seasonal outbreaks. An interaction between age and outbreak was observed (p<0.001) indicating a difference in age effects between circulating virus types; this interaction persisted for seasonal outbreaks only (p<0.001). The outbreaks also differed regarding when the age groups received their diagnosis (p<0.001). A post-hoc analysis showed a tendency for the young age groups, in particular the group 10–19 year olds, led outbreaks with influenza type A H1 circulating, while A H3N2 outbreaks displayed little variations in timing. The validation analysis showed a strong correlation (r = 0.625;p<0.001) between the recorded numbers of clinically and microbiologically defined influenza cases. Our findings demonstrate the complexity of age effects underlying the emergence of local influenza outbreaks. Disentangling these effects on the causal pathways will require an integrated information infrastructure for data collection and repeated studies of well-defined communities.

## Introduction

A thorough understanding of the occurrence and comparative timing of influenza infections in different age groups is important for developing community response and disease control measures, e.g. early social distancing measures, risk communication, and vaccinations (WHO 2009). However, the relationship between age and disease transmission patterns within populations is difficult to measure. Viboud et al (2006) reported that working-age adults are responsible for the between-community transfer of influenza infection during outbreaks [1]. Some studies have attributed the local spread of influenza outbreaks to high attack rates among children and adolescents, suggesting the need to target disease mitigation interventions within this age group [2], [3], [4]. The Houston Family Study reported different age distributions for seasonal H1N1 and H3N2 infections, noting that more than 50% of H1N1 infections were detected among 10–34 year olds [5]. Some studies have identified young children as leading the spread of infection [6], while other studies have identified adolescents and young adults as the age groups most likely to drive local spreads [7]. Other studies have even observed little age-specific difference in the timing of infection onset [8].

Local surveillance is needed to assess community-level influenza activity, as mixing between regions appears to be too weak a variable to infer causality in the direction and timing of spread [9]. The challenge for such surveillance is not to find the causal agent of the disease, but to detect outbreaks and address their proximal and distal causes. Proximal causes of influenza infection include those that influence the probability of exposure to the virus, while distal determinants arise when exposure does not necessarily progress to disease [10]. The age-related impact associated with proximal causes, such as close human-to-human contact patterns and personal hygiene habits in stable communities, can be expected to change slowly over time. In contrast, the age-related impact on the distal causal pathway, reflecting the interplay between the biological virus type characteristics and the immunological status of the host, can be observed in the between-outbreak variations in age-related influenza morbidity.

This study uses an open cohort design to investigate the occurrence of differences between age groups with regard to the proportion of individuals receiving medical care for influenza and their comparative time of diagnosis during outbreaks. The study uses data from an electronic health data repository covering the entire population in a Scandinavian county. Clinical diagnosis of influenza is used as the case definition. Specifically, the aim is to investigate whether age was a determinant for diagnosis of influenza in the county during the period 2005–2010, either alone or in interaction with an epidemic outbreak caused by a particular circulating virus type. A secondary goal was to investigate if age was associated with the within-outbreak point in time of infection onset.

## Methods

The study was performed in Östergötland County (population 427.000) (Table S1) located in South-Eastern Sweden, during the influenza seasons 2005–06 to 2009–10. The number of individuals in this population clinically diagnosed daily with influenza was used as a measure of influenza activity, and the number of days that elapsed from the start of an influenza outbreak to the time for diagnosis was used as a measure of the comparative time of infection onset. The start and end time of each outbreak was defined as eight incident cases during a floating seven–day period.

Östergötland county consists of thirteen municipalities, of which two (Linköping and Norrköping) account for about two thirds of its population. A European highway and the main train connection between Stockholm and Copenhagen run across the county, which, outside urbanized areas, consists mainly of farmland. Employees at several large companies and one university situated in the county also use two local airports for business travel to international destinations. Comparing the demographic characteristics of Östergötland's municipalities with the corresponding statistics for the three metropolitan regions in Sweden (Stockholm, Västra Götaland, and Skåne counties; 108 municipalities), as well as the remaining seventeen counties (169 municipalities) (Information S1), reveals that the demography in Östergötland is quite similar to that of non-metropolitan Sweden. However, the municipalities of Östergötland tend to have a higher share of young people, as well as a lower share of foreign born, compared to the municipalities in other non-metropolitan counties. Within the county, the municipality of Linköping stands out, exhibiting the lowest mean age, the highest education level, and a substantial amount of people commuting there to work.

For those individuals for whom seasonal influenza vaccination is found medically indicated (the elderly and immunosuppressed individuals), the vaccine is administered by a physician during an office visit free of charge. According to public health records, each year about 60% of the population above 64 years of age receives the seasonal influenza vaccine. The general population can get the vaccine for a charge of approximately $45 from their primary care centre. Unlike the seasonal vaccines, during the 2009 A/H1N1 outbreak the pandemic vaccine was provided free of charge to the general population as a part of a national mass vaccination campaign. This campaign was administered in supplementary mass vaccination sites at hospitals and public health clinics throughout the county.

### Ethics Statement

The study design was based on administrative public health databases established for the purpose of systematically and continuously developing and securing the quality of service, and where according to Swedish legislation (SFS 2008:355) personal identification data had been removed from the records.

### Data collection

Two data sources were used for the study. Annual aggregated data on the sex, age, and residence (urban, rural) of the population were collected from Statistics Sweden and grouped into nine age groups (0–9 years, 10–19 years, etc. up to 80+years). Age and sex data from individuals clinically diagnosed with influenza were identified from the data repository connected to the electronic health record systems at Östergötland County Council [11]. The repository collects data from primary care, hospital care, and clinical laboratories. However, data from the clinical laboratories were only available from the period 2009-01-01 to 2010-09-15. Influenza cases were identified by the ICD-10 codes for influenza (J10.0, J10.1, J10.8, J11.0, J11.1, J11.8). For individuals having received an influenza diagnosis at both primary and secondary levels of care, only the latter record was used for the analyses. Data from the microbiological laboratory were not used for case identification, but solely for validation purposes.

### Data analyses

Descriptive statistical methods were applied to the clinical data to help represent influenza activity in the county during the study period. The Relative Illness Ratio (RIR), i.e. the ratio of the percentage of individuals with an influenza diagnosis in a given age group to the percentage of the general population belonging to the same age group, was computed for each age group and outbreak (circulating virus type) using the formula
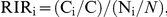
where C_i_ is the number of influenza cases in age group i, C is the number of influenza cases in total, N_i_ is the population in age group i, and N is the total population in Östergötland. RIR values from seasonal and pandemic influenza outbreaks were compared using a method based in normal approximation of the Poisson distribution. The tests were two-tailed, with a 5% risk of type I error.

In the next step of the analysis, a logistic regression analysis was carried out to compute whether the probability for an individual to be diagnosed with influenza was determined by the variables age and outbreak (circulating types of influenza virus); main effects and interactions between these. In this study, the analyses were structured to allow comparisons of coefficients and odds ratios with a neutral reference variable corresponding to a computed average. Two separate analyses were carried out, including and excluding the A pH1N1 outbreak in 2009, respectively, to examine what distinguished the pH1N1 outbreak from the seasonal influenza outbreaks. Finally, the outbreaks (circulating virus types) were tested in pairs to examine interactions between age group and outbreak with regard to the probability of being diagnosed with influenza.

To investigate whether the time of infection onset during outbreaks was determined by age, an analysis of variance (ANOVA) based on the day for diagnosis was performed in the subpopulation having received an influenza diagnosis. Mean differences in time of infection onset were then calculated for each age group. The A pH1N1 outbreak in 2009–10 had two peaks; separate analyses were performed for each of these. Finally, we investigated associations between the mean time of diagnosis and the RIR for the age group during the outbreaks. The correlation between age group effects in the analysis of time of diagnosis and age group regression coefficients in the analysis of proportion of individuals with an influenza diagnosis was calculated for all six outbreak peaks.

The level of statistical significance was set to p<0.05. To denote the strength of correlations, we used limit values suggested by the Cohen Scale [12]. This scale defines small, medium and large effect sizes as 0.10, 0.30, and 0.50 respectively.

In a validation step of the analysis, the case data defined by clinical diagnoses was validated against case data from the microbiological laboratories. In these analyses, both data sets were separately adjusted for week-day effects on care resource utilization. The correlations between the number of cases reported each day in the clinical and laboratory data were analyzed with 0–6 day lag. Also, the age-related risk for receiving an influenza diagnosis was computed from both data sets and compared. The analyses were performed using Minitab Statistical Software version 16.1.1 (Text S1) and reported according to the STROBE statement for observational studies [13].

## Results

**Figure 1:**
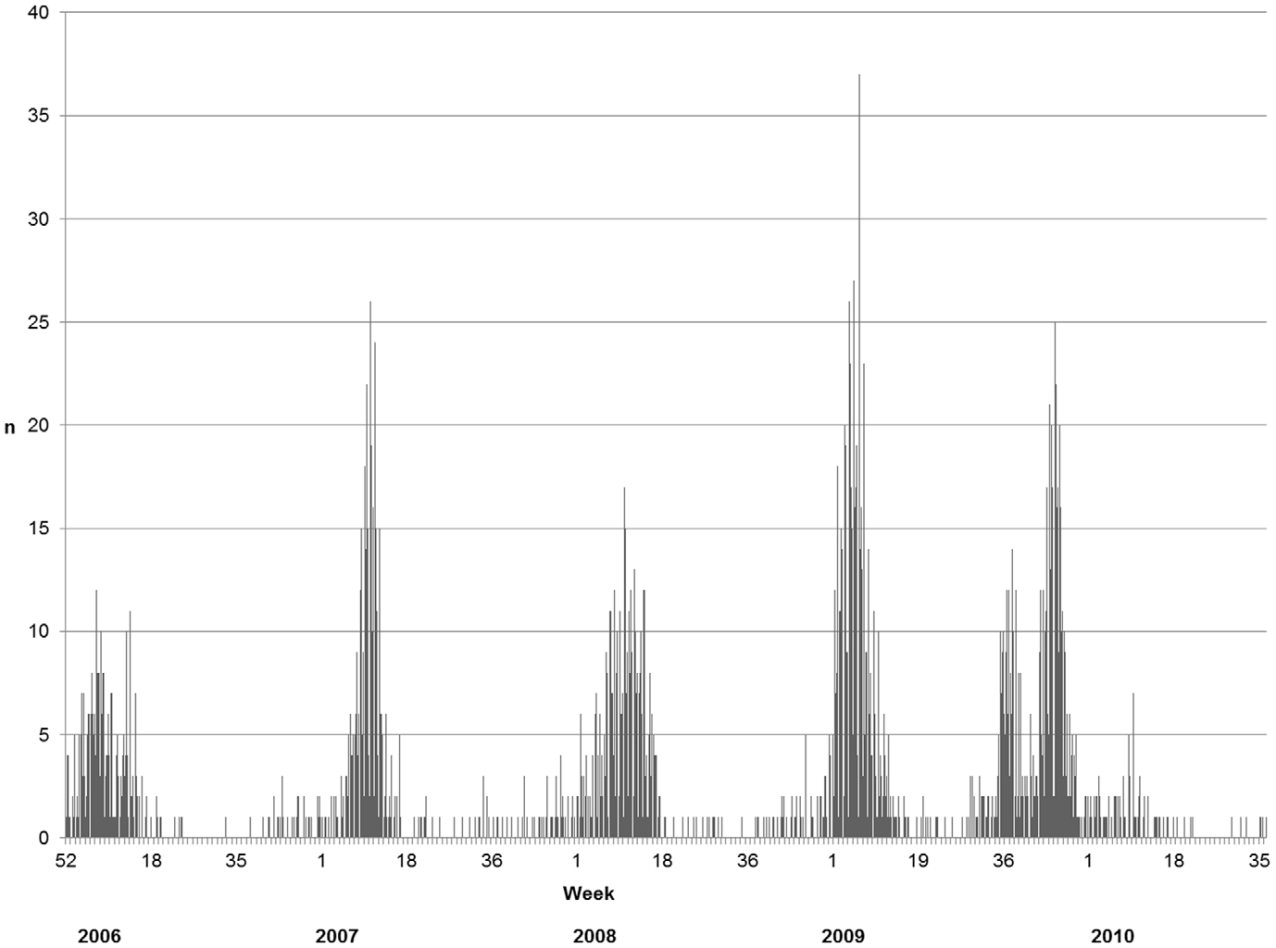
Influenza outbreaks in Östergötland county 2006–2010. Influenza cases (ICD-10 codes 10.0–11.8) per day in Östergötland county 2005–2010. The influenza activity as accumulated into five outbreaks lasting between 2006-01-01–2006-04-20 (circulating virus types B, A/H3 and H1N1), 2007-01-31–2007-04-11 (A/H3N2), 2008-01-21–2008-04-30 (B and A/H1), 2008-12-24–2009-03-30 (A/H3N2), and 2009-08-21–2009-12-22 (A pH1N1).

Five influenza outbreaks with corresponding main circulating virus types were identified (Figure 1). The influenza activity accumulated into five outbreaks lasting between:

- 2006-01-01–2006-04-20 (circulating virus types B, A/H3 and H1N1),
- 2007-01-31–2007-04-11 (A/H3N2),
- 2008-01-21–2008-04-30 (B and A/H1),
- 2008-12-24–2009-03-30 (A/H3N2), and
- 2009-08-21–2009-12-22 (A pH1N1).

The outbreaks differed with regard to intensity, i.e. the risk for residents to receive an influenza diagnosis. The highest intensity was recorded for the A H2N3 outbreak in 2008 (1.44 of the average risk (95% C.I. 1.30–1.60) and second highest intensity during the A pH1N1 outbreak in 2009–10 (1.23 (95% C.I. 1.08–1.40)). The lowest intensity (0.64 (95% C.I. 0.54–0.75)) was recorded for the mixed B, A H3, and A H1N1 outbreak in 2006.

### Age as determinant of receiving an influenza diagnosis

Up to a ten-fold age-group difference in cumulative incidence of influenza cases was observed in the outbreaks recorded during the study period (Table S2). For instance, 2.32 cases per 1000 individuals were diagnosed with influenza in the group 10–19 years old during the A pH1N1 outbreak in 2009 compared to 0.20 cases per 1000 individuals for the age group 70 years and older during the same outbreak. Extending this comparison across all five outbreaks, individuals 30–39 years old demonstrated the highest risk of receiving an influenza diagnosis (1.99 times the average risk; 95% C.I. 1.79–2.22), followed by those 0–9 years of age (1.83 (95% C.I. 1.63–2.05)). The lowest risk was observed for individuals 70–79 years old (0.35 (95% C.I. 0.27–0.46)), and the oldest group 80 years of age and above (0.25 (95% C.I. 0.17–0.37)).

**Figure 2:**
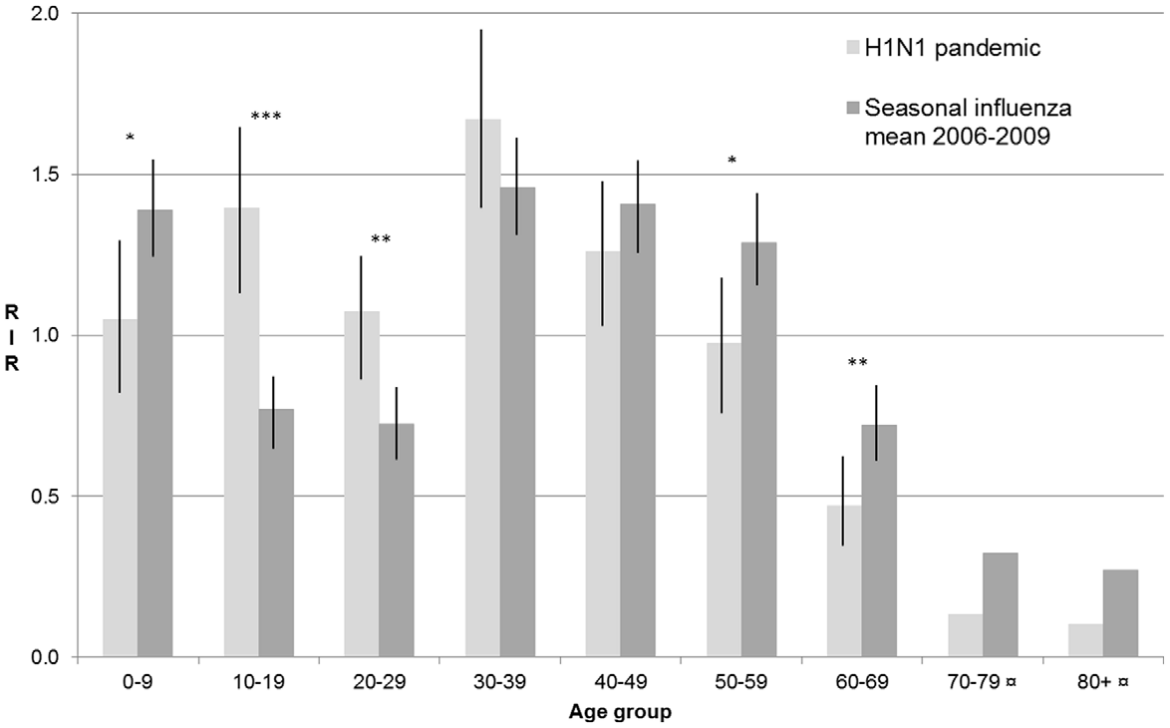
RIR diagrams for pandemic and seasonal influenza outbreaks in Östergötland county 2006–2010. The RIR diagrams (95% Confidence Intervals) represent the A pH1N1 outbreak in 2009 and mean values for the seasonal outbreaks 2006–2010, respectively. * p<0.05 **p<0.01 ***p<0.001 ¤ Too few observations to allow statistical analysis.

**Figure 3:**
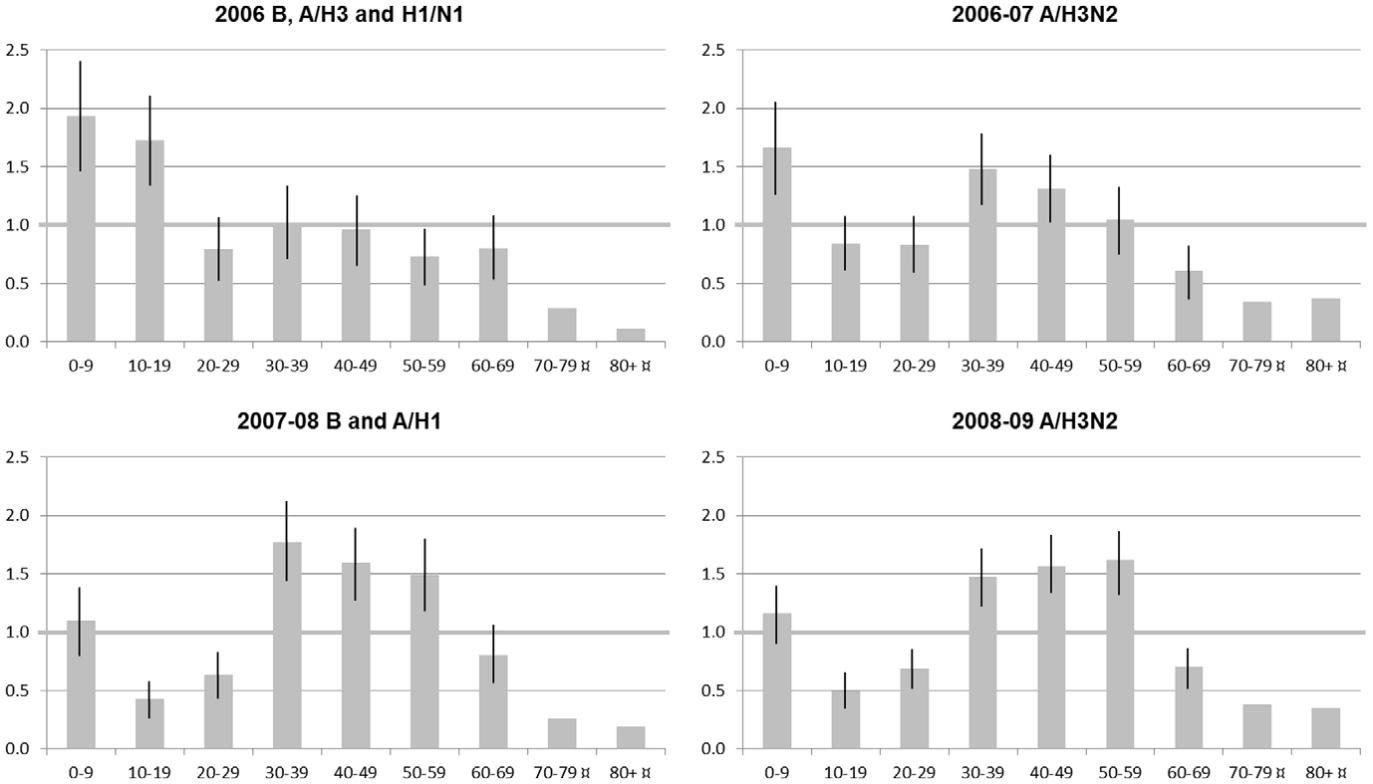
RIR diagrams for seasonal influenza outbreaks in Östergötland county 2006–2009.

RIR-curves comparing the A pH1N1 outbreak in 2009 to the mean for the four seasonal outbreaks are displayed in Figure 2. Larger proportion of influenza cases were attributed to the ages 10–19 (p<0.001) and 20–29 years old (p<0.01) during the A pH1N1 outbreak than during the seasonal outbreaks, while the proportion of cases observed in the age groups 0–9 years (p<0.05), 50–59 years (p<0.05), and 60–69 years (p<0.01) were larger during the seasonal outbreaks. Corresponding curves for each seasonal outbreak are displayed in Figure 3. It is noteworthy that higher- than- expected proportions of cases were distributed to the middle-aged groups (30–39 and 40–49 years) during all seasonal outbreaks except the A H3 and A H1N1 outbreak in 2006.

In the logistic regression analysis that covered all five outbreaks and included combined terms, a statistically significant interaction (p<0.001) between age and outbreak (circulating virus type) was observed, indicating a difference between outbreaks (circulating virus types) regarding age effect on influenza morbidity. However, also when only seasonal influenza outbreaks were included in the analysis, an interaction was observed between age and outbreak (p<0.001). It was thus not the case that the risk associated with an age group was the same during the seasonal outbreaks. A pair-wise post-hoc analysis showed that the interaction between age and outbreak was statistically significant for all but one of the pairs, namely for the A H3N2 outbreak in 2007 and the B and A H1 outbreak in 2008. For all other outbreak pairs, the age effects on proportions of individuals diagnosed with influenza differed between the outbreaks.

### Comparative time for diagnosis in age groups

There was a statistically significant difference between the outbreaks regarding when age groups received a diagnosis in relation to the mean for the outbreak (p<0.001). A post-hoc analysis showed a tendency for the young age groups, in particular the group 10–19 years old to lead the outbreaks with the A H1 type circulating virus (Table 1). The A H3N2 outbreaks displayed little variations in timing, with the age group 30–39 years old leading the outbreak in 2006–07 and the group 10–19 years old leading the outbreak in 2008–09. There was a strong correlation (r>0.5) between the mean time of diagnosis for an age group and its RIR only during the A H3 and A H1N1 outbreak in 2005–06 (Table 2). For all other outbreaks, the correlations were moderate to small.

**Table 1:** Mean time (day) for diagnosis (95% C.I.) for age groups during influenza outbreaks in Östergötland county 2005–2009 with reference to the total mean for the outbreak.

	Outbreak (influenza type)											
	2005–06 (B, A/H3 and H1N1)		2006–07 (A/H3N2)		2007–08 (B and A/H1)		2008–09 (A/H3N2)		2009 (pH1N1), 1st wave		2009 (pH1N1), 2nd wave	
Age (yrs)	n	Mean day (95% C.I)	n	Mean day (95% C.I)	n	Mean day (95% C.I)	n	Mean day (95% C.I)	n	Mean day (95% C.I)	n	Mean day (95% C.I)
**0–9**	66	−13 (−18 –−7)	76	0 (−6–5)	56	−6 (−12–0)	85	1 (−4–6)	11	1 (−10–12)	70	1 (−4–5)
**10–19**	76	−21 (−26–−15)	48	0 (−7–6)	26	−3 (−12–6)	42	−1 (−8–7)	27	−3 (−10–4)	97	−1 (−4–3)
**20–29**	33	−9 (−17–−1)	46	2 (−5–9)	39	−1 (−9–6)	62	1 (−5–6)	33	1 (−6–7)	69	0 (−4–4)
**30–39**	43	3 (−4–10)	82	−3 (−8–2)	106	1 (−4–6)	123	0 (−4–4)	51	−1 (−6–4)	96	2 (−1–6)
**40–49**	42	6 (−1–13)	76	1 (−5–6)	101	−6 (−11–−1)	141	3 (−1–7)	45	−2 (−8–3)	74	0 (−4–4)
**50–59**	31	−8 (−16–1)	58	−1 (−7–5)	89	0 (−5–5)	134	1 (−3–5)	21	2 (−6–10)	64	−1 (−6–3)
**60–69**	30	−7 (−15–2)	31	3 (−6–11)	46	0 (−7–7)	58	5 (−1–11)	17	3 (−6–11)	24	−1 (−8–6)
**70–79**	7	16 (−2–33)	11	−2 (−16–12)	9	0 (−16–16)	19	−3 (−14–8)	3	4 (−17–25)	4	5 (−13–24)
**80+**	2	31 (−2–65)	9	2 (−14–17)	5	15 (−6–36)	13	−6 (−19–7)	1	−4 (−41–32)	3	−5 (−26–16)

**Table 2:** Correlation (95% C.I.) between mean time (day) of diagnosis and RIR for age groups during influenza outbreaks in Östergötland county 2005–2009.

Outbreak (influenza type)											
2005–06 (B, A/H3 and H1N1)		2006–07 (A/H3N2)		2007–08 (B and A/H1)		2008–09 (A/H3N2)		2009 (pH1N1) 1st wave		2009 (pH1N1) 2nd wave	
n	r (95% C.I.)	n	r (95% C.I.)	n	r (95% C.I.)	n	r (95% C.I.)	n	r (95% C.I.)	n	r (95% C.I.)
330	−0.80 (−0.96–−0.30)	437	−0.32 (−0.81–0.43)	477	−0.45 (−0.86–0.31)	677	0.52 (−0.22–0.88)	209	−0.24 (−0.78–0.50)	501	0.03 (−0.65–0.68)

### Validation of clinical case data

The validation analysis, where both data sets were separately adjusted for week-day effects, showed a strong correlation between the number of clinically diagnosed influenza cases per day and the corresponding number of cases verified daily by microbiological analyses during the validation period. The strongest correlation (r = 0.625; p<0.001) was observed between the clinically and the microbiologically verified cases with a 2-day lag. The risk of receiving an influenza diagnosis estimated from the clinical cases and the microbiologically-verified cases showed similar patterns with risk decreasing with age. In both data sets, a statistically significant difference was observed only between the three youngest and the two oldest age categories (Table S3).

## Discussion

We found that the age group-related cumulative incidence of influenza cases differed both between the A pH1N1 and the seasonal outbreaks and in-between the seasonal outbreaks, and that the outbreaks differed with regard to when the age groups received diagnoses. There was modest correlation between the mean time of the diagnosis for an age group and its RIR during outbreaks. These findings exhibit the complexity of age effects on the proximal and distal causes in the emergence of local influenza outbreaks. Regarding the proximal causes, we did not collect data on individual-level social contacts or personal hygiene. However, assuming that the community remains socially stable and that the effect from differences in population immunity remains level over multiple outbreaks, exposure to infectious individuals stands out as perhaps the most important determinant of long-term influenza morbidity in the different age groups. Analogous to previous long-term studies [14], we found that the highest risk of receiving an influenza diagnosis during the 5-year study period was for individuals 30–39 years old. Interpretation of the distal causes suggests a lower degree of preexisting specific immunity, which can help explain overrepresentation of younger age groups among the pandemic cases in comparison with seasonal influenza cases [15]. In 2009, specific immunity from memory T-cells could also have been present in of the population due to shared antigenic epitopes between A pH1N1 virus and recent seasonal influenza A H1N1 viruses and vaccine strains [16]. In our data of the A pH1N1 outbreak in 2009 , we noted (Figure 2) higher relative illness rates among school-age children, adolescents and young adults compared to the seasonal outbreaks. These observations complement previous reports on the seasonal evolution of influenza A virus. Rambaut et al. [17] identified a weaker antigenic drift in H1N1, leading to a global co circulation of multiple H1N1 lineages and weaker A H1N1 bottleneck effects between seasons compared to those of A H3N2. If influenza A H1N1 does preferentially target a younger population, lower antigenic pressure and less-severe bottlenecks in the viral population, are to be expected.

Consistent with a recent Canadian study based on microbiologically verified influenza cases [9], we observed that the A pH1N1 outbreak cases in 2009 peaked earlier among children and youth aged 10–19 years, although the timing of cases was not statistically different for the age groups. Several studies have identified schoolchildren as the drivers of the local spread of influenza, prompting considerations of influenza vaccination for all schoolchildren and the use of school closures to mitigate outbreak effects [18]–[19]. Both our findings and the Canadian results do not support the inclusion of younger school-age children (<9 years) in the lead group for influenza virus transmission during pandemics or seasonal outbreaks. Not all studies agree on the likely benefits of closing schools [20], and our results suggests that the effect of age on timing may be smaller than predicted by previous models. However, a shortcoming of our study is that school closures during and around outbreaks was not taken into regard. This weakness may particularly have influenced the analyses of the age-related timing of influenza morbidity. In addition, it must be considered that the age-related variations in morbidity and timing of diagnosis can have been associated to population demography. A recent study that compared the transmission characteristics of the pH1N1 virus in different countries concluded that countries with higher proportions of children (under 20 years) had higher estimated R_0_ values and morbidity rates [21]. In light of these observations, the role of youth and young adults as potential drivers of seasonal and pandemic influenza outbreaks can still be considered important. Although our results question whether younger school-age children lead epidemic waves of all influenza types, interventions targeting young children may still have an impact on the size of the epidemic.

We observed that larger proportions of influenza cases were attributed to the ages 10–19 and 20–29 years old during the A pH1N1 outbreak than during the seasonal outbreaks, while the proportions of cases observed in the age groups 0–9 years, 50–59 years, and 60–69 years were larger during the seasonal outbreaks. Variability in influenza activity by age in a single community was early noted in a study by Monto et al. [22], where also infection with type A H1N1 was detected at low frequency in adults. Several decades later, we contend that the age-related variability of influenza activity still warrants more and extended studies, and that the single community design remains suitable for investigation of the complex interactions between the proximal and distal causes of influenza morbidity. Although our study population was limited to one community, it is representative for Sweden with regard to basic sociodemographic parameters. The setting is thereby appropriate for study of the influence on influenza dissemination from factors traditionally studied in epidemiology, for instance demographic population structures [21] and social deprivation [23], and also for using novel methods such as densely parameterized community models and cross-validation by simulations [24]–[27].

Our study design still has several important shortcomings. First, the recorded influenza cases reflect only a small subset of the actual symptomatic cases, the majority of which is not expected to seek medical care [28]. Cases requiring admission to the intensive care unit or with a fatal outcome were also not identified. In addition, we have no data on seroconversion in the population during the study period. Seroprevalence studies evaluating the temporal changes in the prevalence of antibodies against the A pH1N1 virus in 2009 can clarify the evolution of the disease amongst different age groups. A British study that applied statistical modeling to evaluate seroprevalence data reported that during the second wave of the A pH1N1 outbreak (September 2009 to February 2010), the cumulative incidence of infection was higher in the age group of 5–14 years, followed by the age group of 1–4 years, and those of 15–24 and 25–44 years [29]. In parallel, Hong Kong researchers tested nearly 15,000 serum samples collected in during the first wave of the 2009 pandemic for antibodies to A pH1N1 [30]. They found that, if these serological data had been available weekly in real time, they would have been able to obtain reliable estimates of influenza morbidity by one week after, one to two weeks before, and three weeks after the pandemic peak for 5–14 year olds, 15–29 year olds, and 30–59 year olds, respectively. They conclude that age-stratified serologic data together with clinical surveillance data could be used to provide reliable real-time estimates of morbidity in an emerging influenza pandemic. The addition of seroprevalence data thus remains a major challenge to future administration of surveillance routines in local community settings.

Apart from differences in the characteristics of the virus, several non-biological factors could account for differences in influenza surveillance data in the pandemic compared with the seasonal influenza outbreaks. These include public health organizations awareness, use of diagnostic methods, public knowledge of influenza influencing healthcare-seeking behavior, and greater sensitivity of health care professionals in pursuing a diagnosis of influenza. A particularly relevant consideration for our study refers to potential differences in vaccination coverage of different age groups of the general population between the pandemic and seasonal influenza periods. In the study county, the elderly were provided vaccine during the seasonal outbreaks, and the results of this study regarding disease incidence for these age groups should therefore be interpreted with care. For most of the A pH1N1outbreak examined in the context of this study, the specific influenza vaccine was not available, and the vaccination coverage of the general population was low. However, the degree in which these parameters could have differentially affected different age groups in the pandemic compared with seasonal influenza periods, and thus confounds our comparative analysis, is difficult to estimate.

In this open cohort study, we found an interaction between age and outbreak, indicating a difference between circulating virus types regarding age effects that persisted for seasonal outbreaks only; in particular, the proportion of cases from the age groups 10–29 years old was larger during the A pH1N1 outbreak in 2009 than during the seasonal outbreaks. In addition, there was a tendency for the young age groups, in particular the group 10–19 years old, to lead outbreaks with influenza type A H1 circulating, while A H3N2 outbreaks displayed little variations in timing. We believe that these findings are generalizable to similar communities with a rectangular age structure. In designing future studies, researchers should carefully consider the role of age within the causal pathway in light of both social and behavioral factors and the biologic characteristics of the circulating influenza virus. The local community environment can modify the interaction between pathogen and host, sometimes influencing both proximal and distal portions of the pathway. For example, social factors, such as socioeconomic status, education and housing/neighborhoods may influence both the exposure to the virus and the probability of developing disease if exposed. Disentangling the age effects in these proximal and distal causal pathways is one of the most important challenges facing infectious disease epidemiologists: this will require an integrated information infrastructure for data collection and repeated studies of well-defined communities. Integration of investigative resources in space and time that enable epidemiological, also including seroconversion data, and prognostic (simulation) studies of the same communities are warranted. Such integrated studies would strengthen the knowledge we have on the occurrence and comparative timing of influenza infections in different age groups as a basis for developing community response and disease control measures.

## Supporting Information

Table S1 Östergötland county population in numbers (percent) displayed by age group, gender, and area of residence.
(DOC)

Table S2 Cumulative incidence of diagnosed influenza cases per 1000 persons population (95% confidence intervals) displayed by gender and level of care during outbreaks 2005–06 to 2009.
(DOC)

Table S3 Validation of clinical case definitions. Odds ratios for receiving an influenza diagnosis relative to an average age class during the A pH1N1 outbreak in 2009 according to laboratory and clinical data sets.
(DOC)

Information S1 Demographic comparison between Östergötland county, the Swedish metropolitan counties, and the rest of Sweden.
(DOC)

Text S1 Details of statistical methods.
(DOC)
