# Supplementary material for: Age as a Determinant for Dissemination of Seasonal and Pandemic Influenza: An Open Cohort Study of Influenza Outbreaks in Östergötland County, Sweden
Source: PLoS One. 2012 Feb 23;7(2):e31746. doi: 10.1371/journal.pone.0031746 (PMC3285651; doi:10.1371/journal.pone.0031746)
Supplement: Table S1 — Östergötland county population in numbers (percent) displayed by age group, gender, and area of residence. (DOC) [file pone.0031746.s001.doc]

**Supporting Table S1.** Östergötland county population in numbers (percent) displayed by age group, gender, and area of residence.

|  | **Urban areas** | | | **Rural areas** | | | **Total** | | |
| --- | --- | --- | --- | --- | --- | --- | --- | --- | --- |
| **Age** | **Women** | **Men** | **Total** | **Women** | **Men** | **Total** | **Women** | **Men** | **Total** |
| 0-9 | 1798 (11) | 18003 (11) | **35101 (11)** | 5544 (10) | 5817 (10) | **11361 (10)** | 22642 (11) | 23820 (11) | **46462 (11)** |
| 10-19 | 18907 (12) | 20060 (13) | **38967 (12)** | 7079 (13) | 7485 (13) | **14564 (13)** | 25986 (12) | 27545 (13) | **53531 (13)** |
| 20-29 | 22099 (14) | 25128 (16) | **47227 (15)** | 4563 (8) | 5304 (9) | **9867 (9)** | 26662 (13) | 30432 (14) | **57094 (13)** |
| 30-39 | 19675 (12) | 21020 (13) | **40695 (13)** | 6080 (11) | 6164 (11) | **12244 (11)** | 25755 (12) | 27184 (13) | **52939 (12)** |
| 40-49 | 20413 (13) | 21479 (14) | **41892 (13)** | 7262 (13) | 7711 (14) | **14973 (13)** | 27675 (13) | 29190 (14) | **56865 (13)** |
| 50-59 | 18571 (12) | 18860 (12) | **37431 (12)** | 7397 (13) | 7635 (14) | **15032 (14)** | 25968 (12) | 26495 (12) | **52463 (12)** |
| 60-69 | 18199 (12) | 17661 (11) | **35860 (11)** | 8137 (15) | 8396 (15) | **16533 (15)** | 26336 (12) | 26057 (12) | **52393 (12)** |
| 70-79 | 12004 (8) | 10073 (6) | **22077 (7)** | 5049 (9) | 4668 (8) | **9717 (9)** | 17053 (8) | 14741 (7) | **31794 (7)** |
| 80-89 | 8678 (6) | 5262 (3) | **13940 (4)** | 3516 (6) | 2380 (4) | **5896 (5)** | 12194 (6) | 7642 (4) | **19836 (5)** |
| 90 + | 1829 (1) | 768 (0) | **2597 (1)** | 796 (1) | 336 (1) | **1132 (1)** | 2625 (1) | 1104 (1) | **3729 (1)** |
| **Total** | **157473 (100)** | **158314 (100)** | **315787 (100)** | **55423 (100)** | **55896 (100)** | **111319 (100)** | **212896 (100)** | **214210 (100)** | **427106 (100)** |
