# Supplementary material for: Age as a Determinant for Dissemination of Seasonal and Pandemic Influenza: An Open Cohort Study of Influenza Outbreaks in Östergötland County, Sweden
Source: PLoS One. 2012 Feb 23;7(2):e31746. doi: 10.1371/journal.pone.0031746 (PMC3285651; doi:10.1371/journal.pone.0031746)
Supplement: Table S2 — Cumulative incidence of diagnosed influenza cases per 1000 persons population (95% confidence intervals) displayed by gender and level of care during outbreaks 2005–06 to 2009. (DOC) [file pone.0031746.s002.doc]

**Supporting Table S2.** Cumulative incidence of diagnosed influenza cases per 1000 persons population (95% confidence intervals) displayed by gender and level of care during outbreaks 2005-06 to 2009.

| **Influenza cases**  **Outbreak year (influenza type/s)** | **Primary care** | | | **Hospital care** | | |
| --- | --- | --- | --- | --- | --- | --- |
|  | **Women** | **Men** | **Total** | **Women** | **Men** | **Total** |
| 2005-06 (A/H3 and H1N1) |  |  |  |  |  |  |
| 0-9 | 1.51 (1.03 – 2.13) | 1.53 (1.06 –2.14) | 1.52 (1.18 – 1.93) | 0 (0 – 0.14) | 0 (0 – 0.14) | 0 (0 – 0.07) |
| 10-19 | 1.10 (0.75 – 1.58) | 1.59 (1.17 – 2.13) | 1.36 (1.07 – 1.70) | 0 (0 – 0.11) | 0 (0 – 0.10) | 0 (0 – 0.05) |
| 20-69 | 0.81 (0.67 – 0.99) | 0.55 (0.43 – 0.69) | 0.68 (0.58 –0.79) | 0.02 (0.01 – 0.07) | 0.03 (0.01 – 0.08) | 0.03 (0.01 – 0.06) |
| 70- | 0.16 (0.05 – 0.37) | 0.18 (0.05 – 0.45) | 0.17 (0.08 –0.31) | 0.06 (0.01 – 0.23) | 0.04 (0.00 – 0.25) | 0.06 (0.01 – 0.16) |
| Total | 0.82 (0.70 – 0.96) | 0.76 (0.64 – 0.88) | 0.79 (0.71 –0.88) | 0.02 (0.01 – 0.06) | 0.02 (0.01 – 0.06) | 0.02 (0.01 – 0.04) |
| 2006-07 (A/H3N2) |  |  |  |  |  |  |
| 0-9 | 1.86 (1.33 –2.53) | 1.59 (1.12 – 2.20) | 1.72 (1.36 – 2.15) | 0 (0 – 0.14) | 0 (0 – 0.13) | 0 (0 – 0.07) |
| 10-19 | 0.97 (0.63 – 1.42) | 0.77 (0.48 – 1.16) | 0.86 (0.64 – 1.15) | 0 (0 – 0.11) | 0.03 (0.00 – 0.20) | 0.02 (0.00 – 0.10) |
| 20-69 | 1.33 (1.14 – 1.54) | 0.88 (0.73 – 1.05) | 1.10 (0.98 – 1.23) | 0.02 (0.01 – 0.07 ) | 0.01 (0.00 – 0.05) | 0.02 (0.01 – 0.04) |
| 70- | 0.38 (0.20 – 0.66) | 0.35 (0.15 – 0.69) | 0.37 (0.22 – 0.57) | 0.03 (0.00 – 0.18) | 0.09 (0.01 – 0.31) | 0.05 (0.01 – 0.16) |
| Total | 1.19 (1.05 – 1.35) | 0.88 (0.76 – 1.02) | 1.04 (0.94 – 1.14) | 0.02 (0.01 – 0.06) | 0.02 (0.01 – 0.06) | 0.02 (0.01 – 0.04) |
| 2007-08 (B and A/H1) |  |  |  |  |  |  |
| 0-9 | 1.45 (0.99 – 2.04) | 1.04 (0.67 – 1.54) | 1.24 (0.94 – 1.61) | 0.05 (0.00 – 0.25) | 0.09 (0.01 – 0.31) | 0.07 (0.01 – 0.19) |
| 10-19 | 0.61 (0.35 –0.99) | 0.36 (0.17 – 0.66) | 0.48 (0.31 – 0.70) | 0 (0 – 0.11) | 0.04 (0.00 – 0.20) | 0.02 (0.00 – 0.10) |
| 20-69 | 1.73 (1.52 – 1.97) | 1.12 (0.95 – 1.31) | 1.42 (1.28 – 1.57) | 0.02 (0.01 – 0.07) | 0 (0 – 0.02) | 0.01 (0.00 – 0.03) |
| 70- | 0.22 (0.09 – 0.46) | 0.30 (0.12 – 0.62) | 0.25 (0.14 – 0.43) | 0.09 (0.02 – 0.28) | 0 (0 – 0.13) | 0.05 (0.01 – 0.16) |
| Total | 1.34 (1.18 – 1.50) | 0.92 (0.80 – 1.06) | 1.13 (1.03 – 1. 23) | 0.03 (0.01 – 0.07) | 0.01 (0.00 – 0.04) | 0.02 (0.01 – 0.04) |
| 2008-09 (A/H3N2) |  |  |  |  |  |  |
| 0-9 | 2.21 (1.64 – 2.91) | 1.47 (1.02 – 2.04) | 1.83 (1.46 – 2.26) | 0 (0 – 0.13) | 0.04 (0.00 – 0.23) | 0.02 (0.00 – 0.12) |
| 10-19 | 0.81 (0.50 – 1.24) | 0.76 (0.47 – 1.17) | 0.78 (0.57 – 1.06) | 0 (0 – 0.12) | 0 (0 – 0.11) | 0 (0 – 0.06) |
| 20-69 | 2.39 (2.13 –2.67) | 1.45 (1.26 –1.67) | 1.91 (1.75 – 2.08) | 0.01 (0.00 – 0.04) | 0.05 (0.02 – 0.10) | 0.03 (0.01 – 0.06) |
| 70- | 0.66 (0.41 – 1.01) | 0.47 (0.23 – 0.84) | 0.58 (0.40 – 0.82) | 0.06 (0.01 – 0.23) | 0.09 (0.01 – 0.31) | 0.07 (0.02 – 0.19) |
| Total | 1.92 (1.74 – 2.11) | 1.26 (1.11 – 1.42) | 1.59 (1.47 – 1.71) | 0.01 (0.00 – 0.04) | 0.05 (0.02 – 0.09) | 0.03 (0.02 – 0.05) |

| 2009 (pH1N1) |  |  |  |  |  |  |
| --- | --- | --- | --- | --- | --- | --- |
| 0-9 | 1.72 (1.23 – 2.35) | 1.76 (1.27 –2.38) | 1.74 (1.39 –2.17) | 0.04 (0.00 – 0.25) | 0.04 (0.00 – 0.23) | 0.04 (0.01 – 0.16) |
| 10-19 | 2.66 (2.07 –3.36) | 2.00 (1.51 –2.60) | 2.32 (1.93 – 2.76) | 0 (0 – 0.12) | 0 (0 – 0.11) | 0 (0 – 0.06) |
| 20-69 | 2.02 (1.79 –2.28) | 1.62 (1.42 – 1.85) | 1.82 (1.66 – 1.99) | 0.05 (0.02 – 0.10) | 0.01 (0.00 – 0.04) | 0.03 (0.01 – 0.05) |
| 70- | 0.25 (0.11 – 0.50) | 0.13 (0.03 –0.37) | 0.20 (0.10 –0.36) | 0 (0.00 – 0.09) | 0.04 (0.00 – 0.24) | 0.02 (0.00 – 0.10) |
| Total | 1.80 (1.63 –1.99) | 1.52 (1.36 –1.70) | 1.66 (1.54 –1.79) | 0.03 (0.01 – 0.07) | 0.01 (0.00 – 0.04) | 0.02 (0.01 – 0.04) |
