# Supplementary material for: Age as a Determinant for Dissemination of Seasonal and Pandemic Influenza: An Open Cohort Study of Influenza Outbreaks in Östergötland County, Sweden
Source: PLoS One. 2012 Feb 23;7(2):e31746. doi: 10.1371/journal.pone.0031746 (PMC3285651; doi:10.1371/journal.pone.0031746)
Supplement: Table S3 — Validation of clinical case definitions. Odds ratios for receiving an influenza diagnosis relative to an average age class during the A pH1N1 outbreak in 2009 according to laboratory and clinical data sets. (DOC) [file pone.0031746.s003.doc]

**Supporting Table S3.** Validation of clinical case definitions. Odds ratios for receiving an influenza diagnosis relative to an average age class during the A pH1N1 outbreak in 2009 according to laboratory and clinical data sets.

|  | **Relative risk for receiving diagnosis during A pH1N1 outbreak in 2009** | | | | |
| --- | --- | --- | --- | --- | --- |
|  | **Laboratory data** | | **Clinical data** | | |
| **Age (yrs)** | **n** | **OR (95% C.I.)** | **Age (yrs)** | **n** | **OR (95% C.I.)** |
| **0-9** | 30 | 2.54 (1.66 - 3.90) | **0-9** | 81 | 1.62 (1.27 - 2.07) |
| **10-19** | 30 | 2.21 (1.44 - 3.39) | **10-19** | 124 | 2.16 (1.74 - 2.68) |
| **20-29** | 33 | 2.29 (1.51 - 3.47) | **20-29** | 102 | 1.67 (1.33 - 2.10) |
| **30-39** | 27 | 2.01 (1.29 - 3.12) | **30-39** | 147 | 2.59 (2.10 - 3.18) |
| **40-49** | 19 | 1.32 (0.81 - 2.15) | **40-49** | 119 | 1.95 (1.57 - 2.42) |
| **50-59** | 15 | 1.12 (0.66 - 1.91) | **50-59** | 85 | 1.51 (1.18 - 1.91) |
| **60-69** | 5 | 0.37 (0.17 - 0.85) | **60-69** | 41 | 0.73 (0.53 - 0.99) |
| **70-** | 1 | 0.07 (0.01 - 0.39) | **70- 79** | 7 | 0.20 (0.10 - 0.40) |
|  |  |  | **80-** | 4 | 0.15   (0.06 -  0.37) |
