# Supplementary material for: Age as a Determinant for Dissemination of Seasonal and Pandemic Influenza: An Open Cohort Study of Influenza Outbreaks in Östergötland County, Sweden
Source: PLoS One. 2012 Feb 23;7(2):e31746. doi: 10.1371/journal.pone.0031746 (PMC3285651; doi:10.1371/journal.pone.0031746)
Supplement: Information S1 — Demographic comparison between Östergötland county, the Swedish metropolitan counties, and the rest of Sweden. (DOC) [file pone.0031746.s004.doc]

# **Supporting Information S1**. Demographic comparison between Östergötland county, the Swedish metropolitan counties, and the rest of Sweden.

The county of Östergötland (Figure S1) is one of Sweden’s 21 counties. Östergötland county consists of thirteen municipalities, of which two (Linköping and Norrköping) account for about two thirds of its population. Figures S2–S11 are box plots that compare the demographic situation in Östergötland’s municipalities with the corresponding statistics for 1) the Swedish metropolitan counties (Stockholm, Västra Götaland, and Skåne; 108 municipalities), and 2) the rest of Sweden

(169 municipalities). The vertical extent of each box represents the interquartile range (IQR) of the concerned distribution; the top and bottom of the box denoting the 75^th^ and 25^th^ percentiles, respectively. The horizontal line in between is the median value. The vertical lines extend to the highest and lowest values, with the exception of outliers and extreme values. Outliers (represented by a ring symbol) are values that are between 1.5 and 3 times the IQR from the nearest end of the box; extreme values (symbolized by an asterisk) are more than 3 times the IQR from the nearest end of the box. The solid horizontal lines superimposed on the box plots represent the median value for all 290 Swedish municipalities. The demographic statistics has been retrieved from official register data at Statistics Sweden, and depicts—except where noted—the situation in 2010.


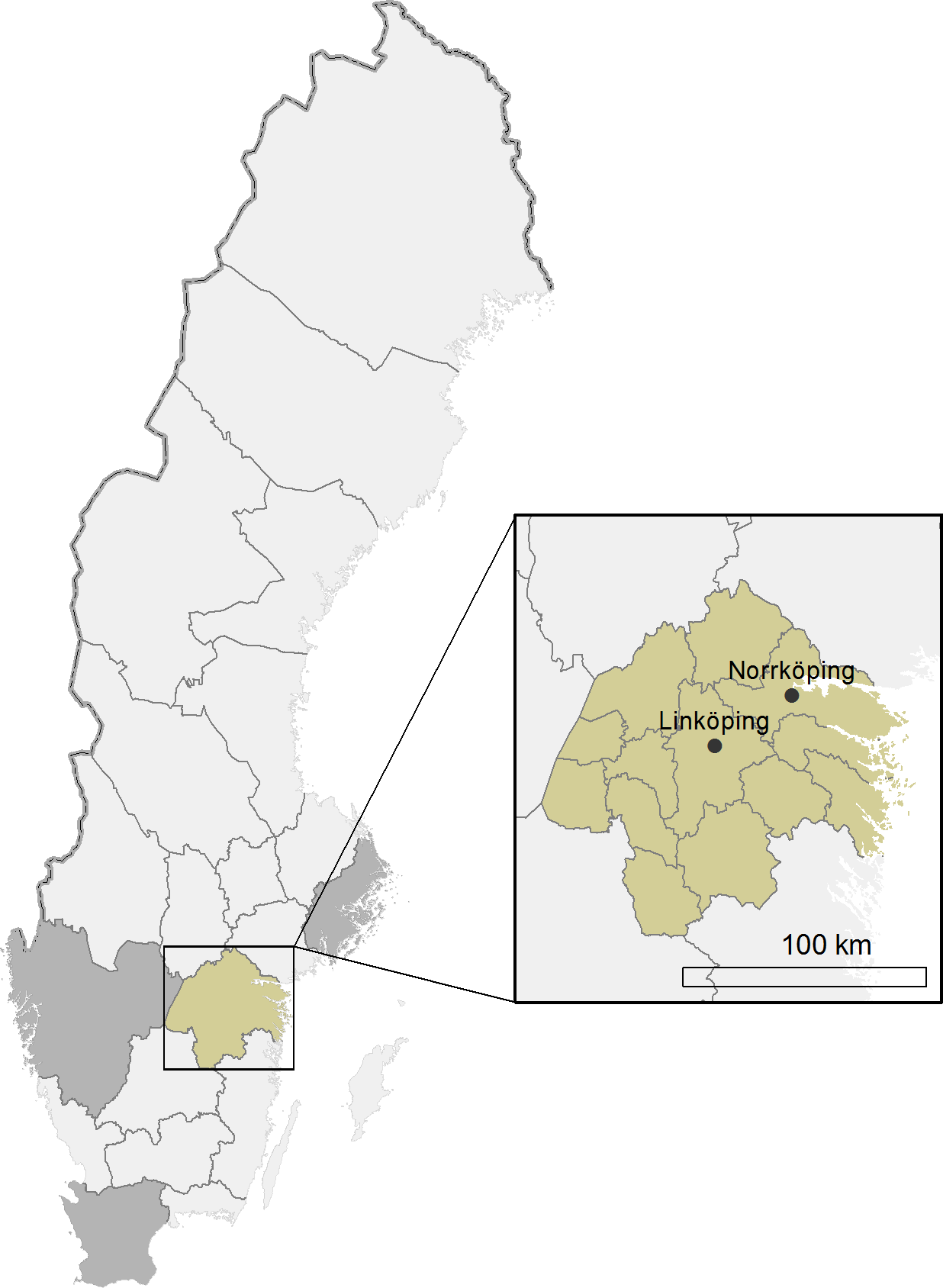


Figure S1: The 21 counties of Sweden. The county of Östergötland is highlighted in yellow, while dark grey is used to symbolize the three metropolitan counties (clockwise from upper right: Stockholm, Skåne and Västra Götaland). The inset map shows the thirteen municipalities of Östergötland county, as well as the location of the two largest urban localities, Linköping and Norrköping.


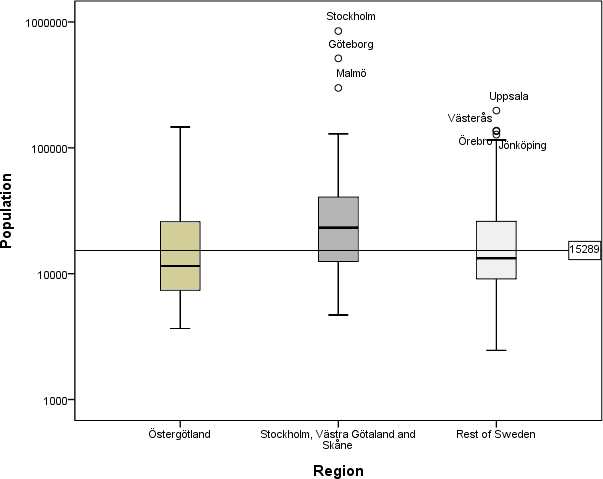


Figure S2. Municipality population size.


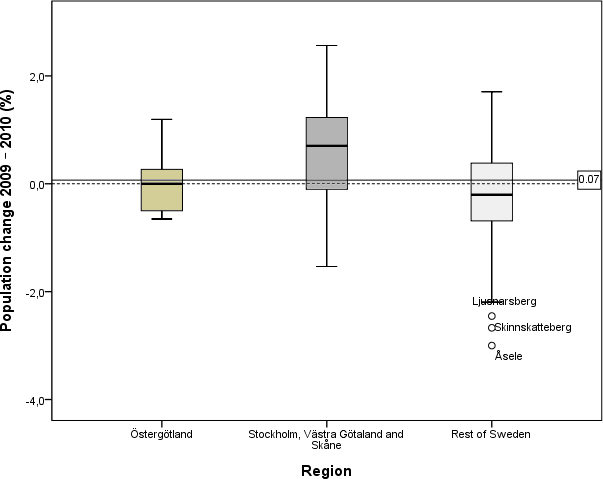


Figure S3. Municipality population change 2009–2010 (%).


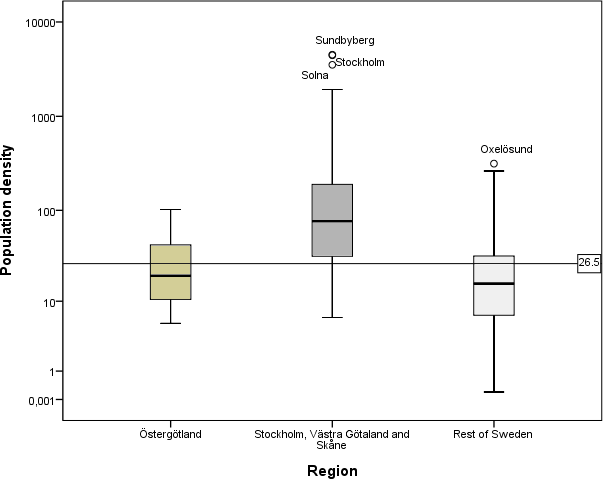


Figure S4. Municipality population density (inhabitants/km^2^).


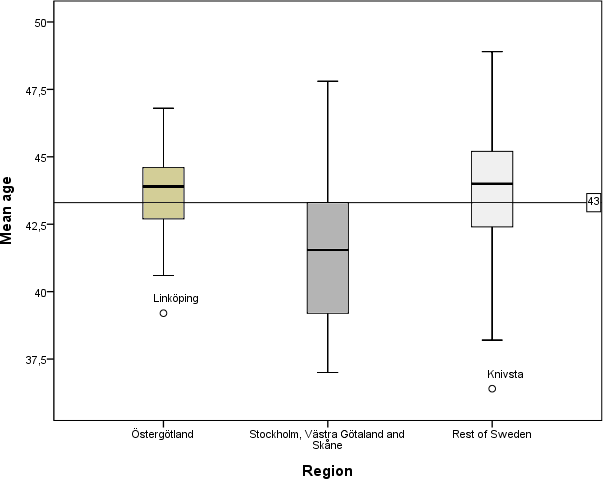


Figure S5. Municipality population mean age.


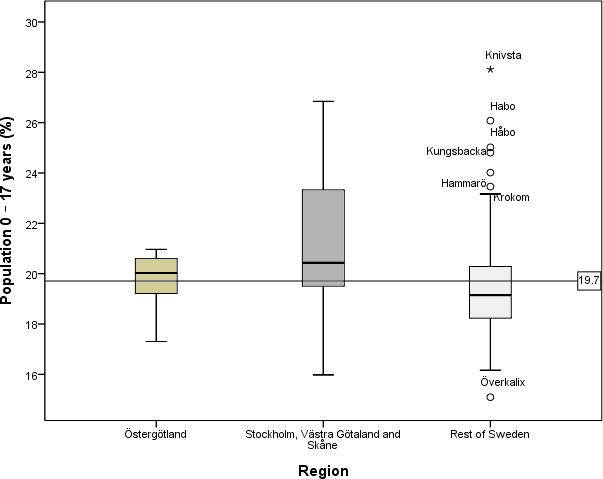


Figure S6. Share of municipality population aged 0–17 years.


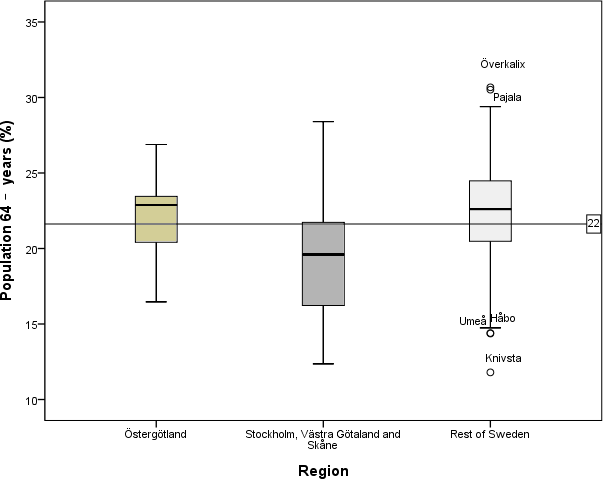


Figure S7. Share of municipality population over 64 years of age.


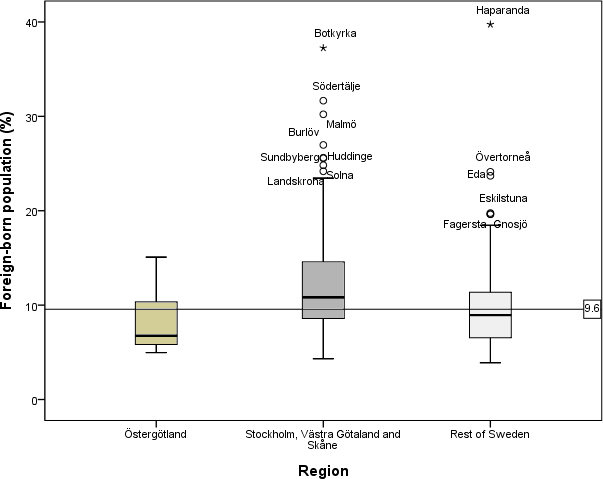


Figure S8. Share of municipality population born in another country.


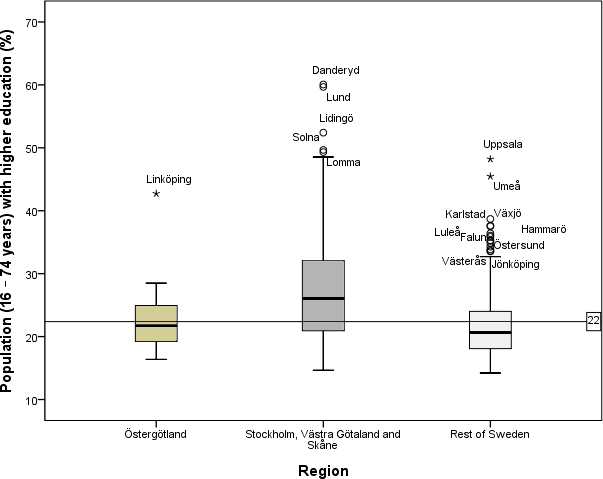


Figure S9. Share of municipality population aged 16–74 years with higher education.


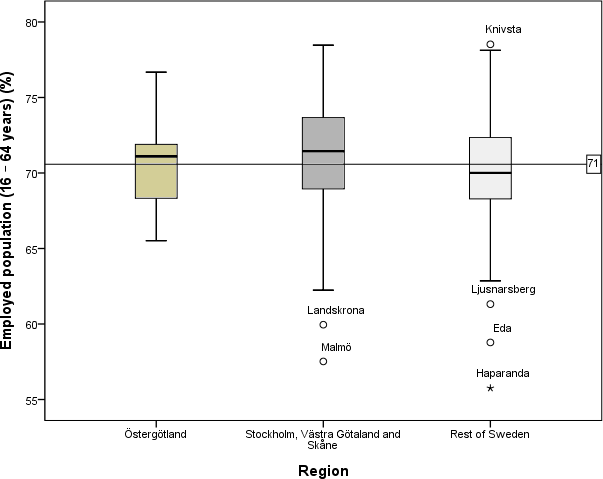


Figure S10. Share of municipality population aged 16–64 years that are employed.


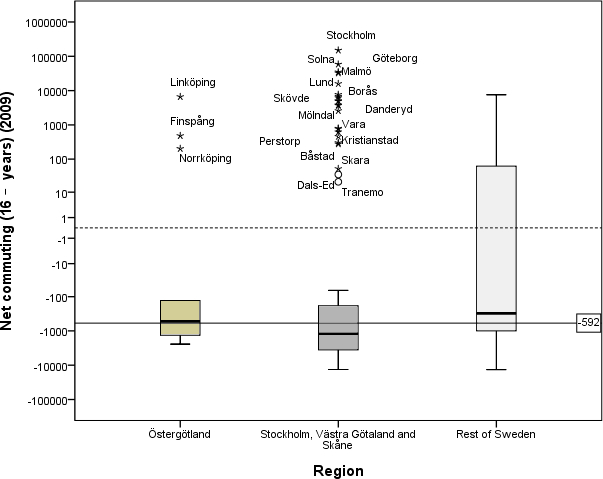


Figure S11. Net commuting among employed (in-commuting - out-commuting) across municipality borders per day.
