# Supplementary material for: Age as a Determinant for Dissemination of Seasonal and Pandemic Influenza: An Open Cohort Study of Influenza Outbreaks in Östergötland County, Sweden
Source: PLoS One. 2012 Feb 23;7(2):e31746. doi: 10.1371/journal.pone.0031746 (PMC3285651; doi:10.1371/journal.pone.0031746)
Supplement: Text S1 — Details of statistical methods. (DOC) [file pone.0031746.s005.doc]

**Supporting Text S1.** Details of statistical methods**.**

**Computations of Relative Illness Ratios**

The formula $RIR_{i}=\frac{\frac{C_{i}}{C}}{\frac{N_{i}}{N}}$ was used, where the *Cs* are variables and the *Ns* are constants. Conditioning on *C* gives $E[RIR_{i}]=E[C_{i}]\times\frac{N}{N_{i}\times C}$ and $Var[RIR_{i}]=Var[C_{i}]\times(\frac{N}{N_{i}\times C})^{2}$. Because *C_i_* acts as the sum of successes in a series of *N_i_* experiments where the probability of success is small and equal in each experiment and the experiments are independent, *C_i_* has approximately Poisson distribution. *C_i_* is the sum of the number of infected men and the number of infected women, meaning that the probability is not exactly the same in each experiment, but because the sum of independent Poisson distributions become Poisson distributed, it is sufficient that the number of infected men and the number of infected women both are Poisson distributed. The variance equals the expected value in the Poisson distribution. Therefore, $Var[C_{i}]$ is estimated with *C_i_* and $Var[RIR_{i}]$ is estimated with $C_{i}\times(\frac{N}{N_{i}\times C})^{2}$. Normal approximation was used for confidence intervals and tests where the number of infected was not too small. The method conditions on *C* and uses approximations. A small simulation study was used to study the consequences of the approximations. The simulation showed that the comparisons become slightly conservative (wider confidence intervals and smaller risk of type-I-error).

**Design of Binary Logistic Regression Analysis**

A logistic regression analysis was carried out to compute whether the probability for an individual to be diagnosed with influenza was determined by the main variables sex, age (9 categories: 0-9 years, 10-19 years, ...., 80 years or more) and outbreak (circulating types of influenza virus); separately or in combinations (interactions) between these. In the analysis, the main class variables were re-coded into indicator variables. To investigate whether the effect of a combination of two or more main variables did not coincide with the combined effects of the main variables separately, interactions between the variables were examined by expanding the model. This was accomplished by adding explanatory variables which were calculated as products of the effects of the explanatory variables.

Regarding the re-coding of the original class variables, the indicator variables can be defined in several ways. One approach is mainly used for comparing one or more groups with a reference group and is called reference coding. This coding is used, for instance, when the objective is to compare two interventions with no intervention at all. In this case it is most natural to use the group not receiving any intervention as the reference group and examine if the effects of the two interventions differ significantly from the untreated group. An alternative method, effect coding, is mainly used when there is no natural reference group, when the objective is to compare interventions or exposures with each other and when no natural "reference group" exists to compare with. What is achieved by effect coding of the variables is that one can compare all the categories within a class variable with the projected medium (average) category. For instance, when effect coding the class variable sex, the effects for men and women are compared with an average sex.

For the present logistic regression analysis, we effect coded the variables that were included in the analysis (sex, age category and outbreak number). The reason was that we found neither sex, age category nor outbreak number having a natural reference group, and therefore each class was compared with a projected medium (average) category. In other words, the effects for men and women were compared with an average sex, the effects of age categories were compared with the average age category and the effects of outbreaks were compared with an average outbreak.

The next step in design of the binary logistic regression was the choice of input variables. First, all variables that were found relevant to include in the model were tested. In our case, the relevant variables were sex, age, outbreak and combination of these (sex*age, sex*outbreak, age*outbreak and sex*age*outbreak). Analysis show that only the interaction age*outbreak is significant. A "problem" that occurs with analysis of class variables (regardless of whether the variables are reference or effect coded) is that when using any regression analysis, one category must always be "left out" in the output. The results (coefficient, odds ratios, significance values, etc.) are thus only shown for n-1 classes, where n is the number of categories. The analysis adjusts the intercept for the reference category, and the other groups are then compared with that category. This issue was simply solved by changing the coding in the coding of groups, running a new analysis, and combining the results by adding the missing row/values to the original analysis. Due to that we used effect coded variables, no values were changed for the other groups. It is easy to verify that no errors were made because it is known that the sum of the coefficients for each class variable (sex, age and outbreak) always adds up to zero. It was also possible to calculate values ​​for the missing group from results in the original analysis, but that was found slightly more complicated. The issue mentioned in this paragraph is thus no calculation problem, but rather a consequence of how the results are presented as default of a regression analysis in most statistical software.

**Week day effect**

We think that what we observe one day consist of three components, the actual level that day (including that only a constant fraction of the infected also is expected to make a visit), the day of week effect and a random component. At day *i*, we have $Y_{i}=L_{i}\times D_{i\mathrm{mod}7}\times E_{i}$ where the capitals are (in order) observed value, level, day of week-effect (numbered 0 to 6) and random component. The day of week-effects must average to 1 over all seven day of week-effects and the random component is positive with expectation 1.

For a longer series, we assume that the mean of Mondays should equal the mean of Tuesdays etc if there were no day of week-effects. Adjusting for day of week-effects means that the observed value at day *i* should be adjusted by dividing with $D_{i\mathrm{mod}7}$, that is $A_{i}=Y_{i}/D_{i\mathrm{mod}7}$ where $A$ is the adjusted level. Therefore, we must estimate $D_{i\mathrm{mod}7}$. We now switch to name the means and day of week effects by week day (truncated to 2 letters) instead of using the subscript *i* mod 7.

The idea is to choose such that the$Ds$average to 1 and that . This is done in two steps.

Step 1: find preliminary effects which do have correct internal relation but not correct level.

Because is the same as which gives when no mean

is zero it can be seen that $D_{Mo}$ is proportional to the reciprocal of $\bar{Y}_{Mo}$ etc. The preliminary estimates, $C$, are

etc.

Step 2: adjust the levels to find estimates of the day of week-effects by keeping the relation between the $Cs$while scaling to an average of 1. This gives $C_{Mo}=C_{Mo}/\bar{C}=\frac{C_{Mo}}{(C_{Mo}+C_{Tu}+...+C_{Su})/7}$etc as estimates of the$Ds$.
